# Effect of Bacterial Nanocellulose with Chemisorbed Antiseptics on Alveolar Bone Repair in Rats Undergoing Bisphosphonate Therapy

**DOI:** 10.3390/pharmaceutics17010024

**Published:** 2024-12-26

**Authors:** Marcelo Matos Rocha, Valesca Sander Koth, Marcela Wiltgen Jeffman, Fernanda Gonçalves Salum, Josiane de Almeida, Karina Cesca, Karen Cherubini

**Affiliations:** 1 Post-Graduate Program in Dentistry, School of Health and Life Sciences, Pontifical Catholic University of Rio Grande do Sul (PUCRS), Porto Alegre 90619-900, RS, Brazil; marcelo.rocha86@edu.pucrs.br (M.M.R.); valesca.koth@edu.pucrs.br (V.S.K.); marcela.jeffman@edu.pucrs.br (M.W.J.); fernanda.salum@pucrs.br (F.G.S.); 2 Department of Endodontics, University of Southern Santa Catarina (UNISUL), Tubarão 88704-900, SC, Brazil; josiane.silveira@animaeducacao.com.br; 3 Department of Chemical Engineering and Food Engineering, Federal University of Santa Catarina (UFSC), Florianópolis 88040–900, SC, Brazil; karina.cesca@posgrad.ufsc.br

**Keywords:** bacterial cellulose, nanocellulose, bone tissue regeneration, graft

## Abstract

**Objectives**: This work investigated the effect of bacterial nanocellulose (BNC) alone or with chemisorbed chlorhexidine or povidone-iodine on post-tooth extraction repair in rats undergoing bisphosphonate therapy. **Methods**: Forty Wistar rats were treated with zoledronic acid, subjected to tooth extractions and allocated into groups according to the material inserted in the post-extraction socket: (1) BNC (*n* = 10); (2) BNC/Iodine (*n* = 10); (3) BNC/Chlorhex (*n* = 10); (4) Control (*n* = 10). Maxillae were dissected and macro- and microscopically analyzed. **Results**: Oral lesion frequency on macroscopic examination did not differ between the groups, whereas it was larger in the BNC/Iodine group compared to the BNC/Chlorhex and Control. BNC/Chlorhex had significantly more connective tissue than did BNC but did not differ from the BNC/Iodine and Control. Epithelium, vital bone, non-vital bone, tooth fragment and inflammatory infiltrate did not significantly differ between the groups. BNC/Iodine showed greater CD31 immunostaining compared to BNC and the Control. Myeloperoxidase staining did not differ between the groups, and scanning electron microscopy analysis showed similar characteristics in all groups. **Conclusions**: BNC with chemisorbed povidone-iodine is associated with increased vascularization in post-extraction wounds of rats undergoing bisphosphonate therapy, whereas BNC with chemisorbed chlorhexidine improves connective tissue formation. BNC works as an effective carrier for the antiseptics tested.

## 1. Introduction

Bisphosphonates are indicated to treat bone resorption diseases such as osteoporosis, bone metastases of solid tumors (breast, lung, prostate) and multiple myeloma [1,2]. Since 2003 [3], the literature has reported cases of medication-related osteonecrosis of the jaws (MRONJ), first caused by bisphosphonates and later on involving other drugs such as denosumab and antiangiogenics [2,4]. MRONJ is mostly triggered by dentoalveolar surgery, including tooth extraction and dental implant placement. Nevertheless, it can also be related to periodontitis, endodontic infection and mechanical trauma, or it can even be spontaneous [2,5,6,7]. Even though bone metabolism inhibition is considered a major causal factor for MRONJ [4], the role of oral microbiota has also been pointed out as crucial for the lesion’s development [4,8].

There is significant morbidity and quality of life impairment in patients with MRONJ, and these individuals still do not show a satisfactory response to available therapies [2,4]. Conventional approaches such as antimicrobials and surgical interventions, or alternative ones such as laser therapy, hyperbaric oxygen therapy, ozone therapy, platelet-rich plasma and pentoxifylline/tocopherol have been used [2,9,10]. The results are variable, from failure to success, depending on specific conditions of the patient, the patient’s disease, the drug causing osteonecrosis and the chosen therapy itself, with no efficacious protocol so far [2,4,11].

Chlorhexidine and povidone-iodine have shown effectiveness in controlling biofilm-based infections, especially in chronic wounds [12,13,14]. Chlorhexidine is the gold-standard topical antiseptic and integrates into the protocol treatment recommended for MRONJ [2,15]. Povidone-iodine, in turn, is an iodophor, formed by molecular iodine (I_2_), which is physically intercalated in the helix of the polyvinylpyrrolidone macromolecule, a solubilizing polymer carrier. It is a broad-spectrum microbicide for gram-positive and gram-negative bacteria, fungi, mycobacteria, chlamydia, viruses and protozoa [16,17].

More recently, bacterial nanocellulose (BNC) membranes have been used in the medical/dental field [18,19] as wound dressing, scaffold for cell cultivation and also carriers for controlled drug delivery [20]. BNC is a natural cellulose synthesized by gram-negative bacteria of the genera *Komagataeibacter (Gluconacetobacter)*, *Agrobacterium*, *Achromobacter*, *Enterobacter*, *Rhizobium*, *Pseudomonas*, *Salmonella*, *Azotobacter* and *Alcaligenes*, as well as gram-positive bacteria *Sarcina ventriculi* and *Rhodococcus* [19]. It is characterized as a biopolymer that stands out as a three-dimensional scaffold in the field of tissue engineering [21,22]. The structure comprises ribbon-like nanofibers, approximately 100 nm wide, which are made up of bundles of cellulose microfibrils, 2 to 4 nm in diameter [21]. This network of cellulose nanofibers exhibits excellent biocompatibility and mechanical properties, with high crystallinity and high water retention capacity [20,23,24]. The structure is similar to that of the collagen membrane, which is the most used material in guided bone regeneration [22]. Studies confirm that various cells can grow in the presence of BNC, favoring the regeneration of body components, such as skin, bones, cartilage, nerves, heart and blood vessels [20,25]. Considering the challenging management of MRONJ and the properties of BNC, the present study aimed at investigating the effect of BNC membranes alone or with chemisorbed chlorhexidine or povidone-iodine on tooth post-extraction alveolar bone repair in an animal model undergoing bisphosphonate therapy.

## 2. Materials and Methods

### 2.1. Sample Characterization

This study was approved by the Ethics Committee on Animal Use of Pontifical Catholic University of Rio Grande do Sul (CEUA-PUCRS), protocol #9994. The procedures followed the guidelines of the National Council for Animal Experimentation Control (CONCEA) and Animal Research: Reporting of in vivo experiments (ARRIVE) [26]. The sample was composed of 40 adult female Wistar rats (*Rattus norvegicus*), 70 days old and weighing between 250 and 300 g at the beginning of the experiment. The animals were housed in microisolators with filtered air, controlled humidity and temperature (23 ± 1 °C) and light-dark cycle of 12 h, with 300 lux in the room and 60 lux inside the cages. Each cage housed at most four animals. Filtered water and food [Nuvilab-Cr1 (Nuvital, Colombo, PR, Brazil)] were given ad libitum. Cleaning and changing the cages were carried out according to the protocol of the facility center (CEMBE/PUCRS). Additionally, the cages were equipped with enriching environmental tools such as PVC pipes and wood blocks. All animals were maintained under the same standardized conditions aimed at controlling stress factors. The choice for single-sex sample was based on ethical concerns (sample size) and previous studies with a similar paradigm which used female rats [27,28,29].

After a 10-day period of acclimatization to the environmental conditions, the animals were treated with zoledronic acid (Eurofarma, Itapevi, SP, Brazil), given intraperitoneally (IP) at 0.3 mg/kg/week for 35 days (5 weeks, 5 doses) [27,28,29]. Afterwards, tooth extractions were performed and the animals were randomly allocated into groups according to the material inserted in the post-extraction socket: (1) BNC group: 10 rats receiving BNC membranes; (2) BNC/Iodine: 10 rats receiving BNC membranes with chemisorbed 10% povidone-iodine; (3) BNC/Chlorhex: 10 rats receiving BNC membranes with chemisorbed 0.12% chlorhexidine digluconate; (4) Control group: 10 rats with no material inserted in the post-extraction socket. Zoledronic acid was given for another 25 days (3 doses) leading to a total of 8 doses (60 days of treatment).

### 2.2. Bacterial Nanocellulose Production

The bacterial strain used for BNC production was *Komagataeibacter hansenii* ATCC 23769, acquired from the Collection of Tropical Culture of André Tosello Foundation (Campinas, SP, Brazil). The inoculum was produced three days before starting the experiment. Before bacterial strain inoculation, mannitol medium made up of 25 g mannitol, 5 g yeast extract and 3 g peptone diluted in 1 L of distilled water, with pH adjusted to 6.6, was sterilized in an autoclave for 20 min at 121 °C. An inoculum-stock solution was obtained and added to the culture medium base at 10% (*v*/*v*) in 96-well culture plates, allowing the production of several BNC membranes. The plates were maintained in static culture at a temperature of 25 °C for seven days. Afterwards, the BNC membranes that grew in the liquid–air interface of the culture well were removed to start the purification process.

The BNC membranes were transferred to a flask containing 0.1 M NaOH, where they were allowed to stand for 24 h at 50 °C, to remove bacteria and/or residues retained in the nanofiber network. Next, the BNC membranes were washed several times in distilled water until they reached pH 7, and they were then sterilized by autoclaving for 20 min at 121 °C. Afterwards, the nanofibers were subjected to an oxidation process to make them chemically more susceptible to the incorporation of 0.12% chlorhexidine digluconate (Rioquímica, São José do Rio Preto, SP, Brazil) and 10% povidone-iodine (Vic Pharma, Taquaritinga, SP, Brazil). Once production was completed, the BNC membranes were kept at 4 °C until used in the experiments.

### 2.3. Surgical Procedures

After 35 days of zoledronic acid administration, extractions of the three right upper molars of the animals were performed. Surgical procedures were under anesthesia with a mixture of ketamine hydrochloride (100 mg/kg; Syntec, Cotia, SP, Brazil) and xylazine hydrochloride (10 mg/kg; Syntec), administered IP. The teeth were extracted using a 3s carver for dislocation and forceps (Quinelato/Schobell Industrial Ltd., Rio Claro, SP, Brazil) of compatible size and adapted to the upper molars of the animal model. Immediately after the extractions, the groups BNC, BNC/Iodine and BNC/Chlorhex received the respective previously prepared BNC membranes, whereas the Control group did not receive any BNC. For postoperative analgesia, dipyrone (200 mg/kg) was administered subcutaneously. After tooth extractions, the animals were kept on zoledronic acid treatment.

### 2.4. Euthanasia

Twenty-five days after the tooth extractions (60 days after starting zoledronic acid treatment), the animals were killed by anesthetic overdose with IP administration of ketamine hydrochloride (300 mg/kg; Syntec, Cotia, SP, Brazil) and xylazine hydrochloride (30 mg/kg; Syntec).

### 2.5. Macroscopic Evaluation

**Figure 1:**
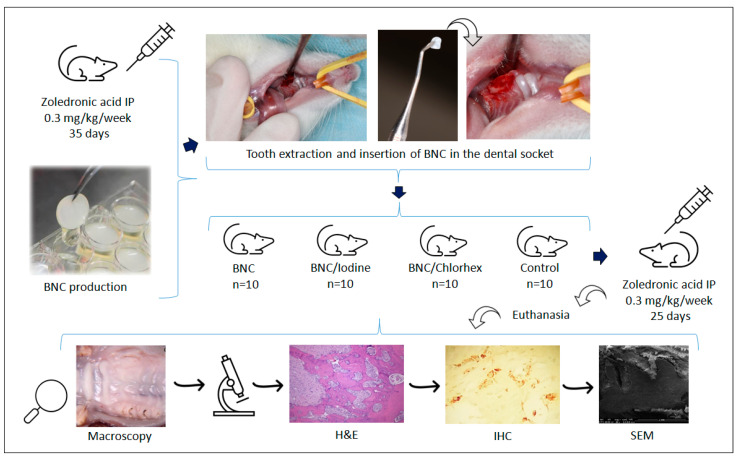
Flow-diagram of the steps of the experiment. After 35 days of treatment with zoledronic acid, the rats were subjected to tooth extractions and insertion of bacterial nanocellulose (BNC) in the tooth socket. The groups BNC, BNC/Iodine, BNC/Chlorhex and Control were kept under zoledronic acid treatment for another 25 days. Next, the animals were euthanized, and maxillae dissected for macroscopic and microscopic analyses. Chlorhex = chlorhexidine; H&E = hematoxylin and eosin; IHC = immunohistochemistry; SEM = scanning electron microscopy.

The maxillae were dissected and macroscopically analyzed. This analysis was conducted by one observer, through visual inspection with the aid of a 12× magnifying glass, a dental explorer No. 5 (Duflex, SS White, Rio de Janeiro, RJ, Brazil) and a periodontal probe. The criterion used was presence/absence of loss of mucosal integrity [27], indicating total coaptation or non-closure of the surgical wound. When there was no complete healing, the area of oral lesion was measured with a periodontal graduated probe. The sequence of procedures with the animal model is illustrated in Figure 1.

### 2.6. Specimen Processing

Immediately after euthanasia, the maxilla was dissected and immersed in an identified flask containing 10% buffered formalin. Next, a fragment comprising the area of tooth extraction was cut and then further divided into two small fragments. The osteotomy was performed with the aid of a 19 × 0.20 mm double-faced total diamond disk (American Burrs, Porto Alegre, RS, Brazil), at low-speed and with irrigation. This segment was cut in the middle, in the coronal direction and divided into two small fragments (≅1.0 cm × 0.7 cm × 0.5 cm each), both containing the tooth extraction area on the cutting surface, to be microscopically analyzed. These two fragments were returned to 10% buffered formalin for 24 h and subsequently subjected to histological processing. Two specimens from each group were fixed in 2% glutaraldehyde and destined for scanning electron microscopy (SEM) processing.

### 2.7. Histological Processing

The specimens were decalcified in 10% nitric acid for about 8 h. After confirming that the decalcification process was complete, they were subjected to routine histological processing and embedded in paraffin. Three histological slides were obtained for each animal, one of them with 4 µm thick tissue sections, and the other two with 3 µm thick sections. The 4 µm thick sections were stained with hematoxylin and eosin (H&E) using the standard technique, and the 3 µm thick sections were subjected to immunohistochemistry (IHC).

### 2.8. Immunohistochemistry (IHC)

The 3 µm thick sections were processed using a Dako Autostainer Link 48 (Agilent-Dako, Santa Clara, CA, USA) with Dako EnVision FLEX+ detection system (Dako, Glostrup, Denmark). Antigen retrieval was with EnVision FLEX Target Retrieval Solution at 97 °C for 20 min and endogenous peroxidase blocking with EnVision FLEX Peroxidase-Blocking Reagent. The slides were incubated for 30 min at room temperature with the primary antibodies anti-CD31 (clone JC70A, Dako) and anti-myeloperoxidase (polyclonal, Dako) at 1:50 dilution in diluent with background reducer components (Dako). Advanced HRP Kit (Dako) was used to amplify the signal, and the reaction was revealed with the chromogen diaminobenzidine (DAB, Dako). Counterstaining was with Harris hematoxylin, and coverslips were mounted with Entellan (Merck Millipore, Darmstadt, Hesse, Germany). Specimens of vermiform appendix and bone marrow were positive controls, respectively, for CD31 and myeloperoxidase. Samples of the study processed without the primary antibodies were the negative controls.

### 2.9. Histological Analysis

Histological images were captured with an Olympus BX-43 microscope (Olympus, Tokyo, Japan) connected to a digital camera using a 10× objective for H&E and a 20× objective for IHC. Four fields in H&E and 3 fields in IHC were captured per slide, in a standardized way (area of tooth extraction, from left to right, top to bottom, in a clockwise direction). In H&E, the entire tooth extraction area was included. In IHC, the zone with greater staining in the tooth extraction area was first localized, and 3 fields were then captured in this area. Images were analyzed in the Image Pro Plus software 4.1 (Media Cybernetics, Silver Spring, MD, USA). Histological analysis was performed by an examiner previously calibrated and blinded to the group to which each image belonged. Calibration consisted of analyzing a series of 20 images, in duplicate, at two different times. The results of these analyses were tested with the intraclass correlation coefficient, which showed r > 0.7. In the H&E images, vital bone, non-vital bone, fibrous connective tissue, inflammatory infiltrate, epithelial tissue and tooth fragment were quantified. The reading was performed by using the manual point counting technique in the Image Pro-plus, applying a grid of 660 points [27,28,30]. Each point in the grid was classified by the observer according to the variable to which it was superposed. By clicking the mouse, the observer gives the information to the software, and the results are expressed as number of points for each variable. Variables were quantified in the four fields captured from each histological slide. The IHC images were analyzed by the semi-automated segmentation technique in the Image Pro-plus software [30] quantifying the positively stained area. In this technique, the observer selects one point stained in brown color and the software automatically selects and quantifies the whole stained area in the image.

### 2.10. Scanning Electron Microscopy (SEM) Process and Analysis

After fixation in glutaraldehyde, the specimens were subjected to three 30-min washes in 0.2 M sodium phosphate buffer and distilled water and dehydrated in acetone baths (30, 50, 70, 90 and 100%). For drying, they were immersed in absolute ethanol and taken to the drying chamber at the critical point, according to the instrument’s protocol. The samples were removed from the chamber and mounted on stubs. The samples were sputter-coated with gold (Dentom Vacuum Desk, São Paulo, Brazil) to form a conductive gold layer and were scanned using an XL 30 scanning electron microscope (Phillips, Eindhoven, The Netherlands). The images were first captured at 80× for screening the tooth extraction area, and then the areas of interest were subsequently captured at 500×, 1000× and 5000×. A chemical analysis to evaluate mineral content of the alveolar bone was performed by using energy dispersive spectroscopy (EDS).

### 2.11. Statistical Analysis

Data were analyzed by means of descriptive and inferential statistics. Descriptive statistics included mean, standard deviation, median, 25th percentile, 75th percentile and mean rank, which were presented in graphs and tables. Lesion frequency on the macroscopic examination was compared between groups with Fisher’s exact test. Parametric data were analyzed with ANOVA complemented by Tukey’s multiple comparison test or with Kruskal–Wallis complemented by Dunn’s multiple comparison test. Variable correlations were tested with Pearson’s correlation coefficient. Analyses were carried out in SPSS 21.0 (IBM Corp., Armonk, NY, USA), at the significance level of 5%. Graphs were constructed in GraphPad Prism 9.0.

## 3. Results

### 3.1. Macroscopic Analysis

**Figure 2:**
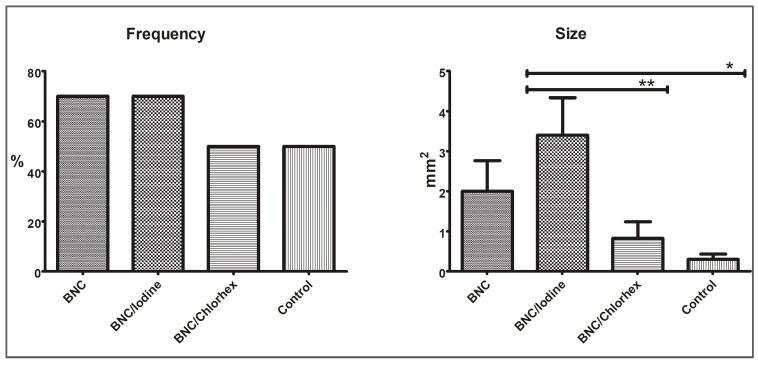
Macroscopic analysis: frequency (%) and size (mm^2^) of the oral lesion. Frequency: *p* = 0.714 (Fisher’s exact test, α = 0.05); Size: * *p* = 0.008; ** *p* = 0.036 (ANOVA, Tukey’s multiple comparison test). BNC = Bacterial nanocellulose; Chlorhex = chlorhexidine.

When comparing the presence/absence of oral mucosal lesions between the groups, the frequency of the oral lesion on macroscopic examination of the maxillae did not differ significantly between the BNC (seven rats with lesion), BNC/Iodine (seven rats with lesion), BNC/Chlorhex (five rats with lesion) and Control (five rats with lesion) groups (Fisher’s exact test, *p* = 0.714, Figure 2). However, the size of the lesions was significantly larger in the BNC/Iodine group (mean = 3.4 ± 2.96 mm^2^) compared to the BNC/Chlorhex (mean = 0.82 ± 1.30 mm^2^) and Control (mean= 0.30 ± 0.40 mm^2^) groups (ANOVA, *p* = 0.008) but did not significantly differ from the BNC animals (mean = 2.0 ± 2.42 mm^2^). The other groups did not significantly differ from each other (*p* > 0.05, Figure 2).

### 3.2. Histological Analysis—H&E

**Figure 3:**
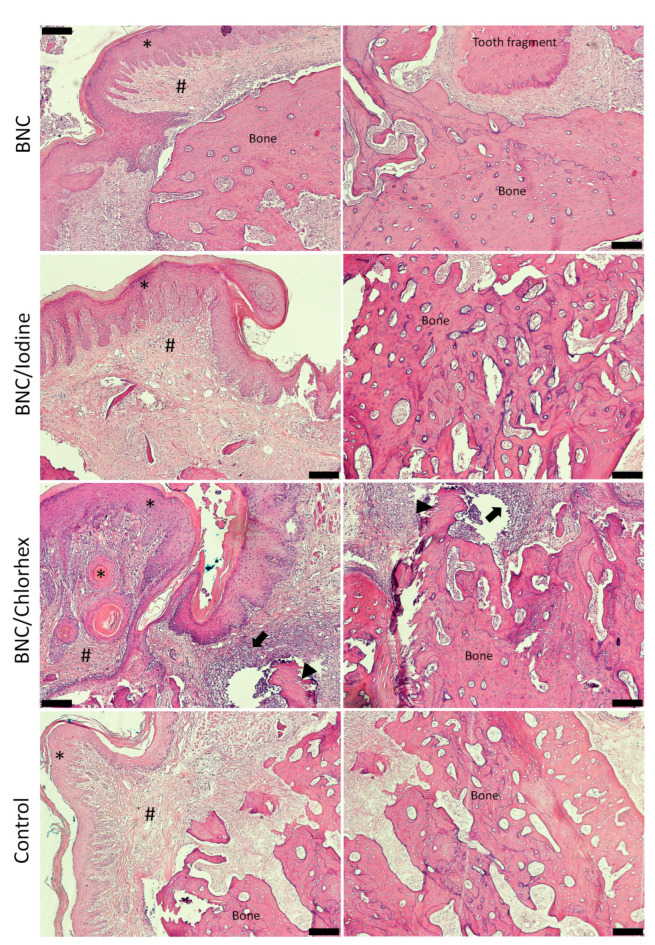
Histological appearance with hematoxylin and eosin staining (H&E, 200×). Epithelium (*); connective tissue (#); inflammatory infiltrate (arrow); non-vital bone (arrowhead); vital bone (Bone). The right column in the figure shows the same sample in the left column with emphasis on the bone area/tissue. Scale bar = 200 µm. BNC = bacterial nanocellulose; Chlorhex = chlorhexidine.

The BNC/Chlorhex group had significantly more connective tissue (median = 191.88 points) than the BNC group (median = 131.25 points) (Kruskal–Wallis, Dunn’s test, *p* = 0.005) but did not differ from BNC/Iodine (median = 141.00 points) and Control (median = 165.75 points). Epithelium, vital bone, non-vital bone, tooth fragment and inflammatory infiltrate did not significantly differ between the groups (Kruskal–Wallis, Dunn’s multiple comparison test, α = 0.05, Table 1, Figure 3).

### 3.3. Correlations

Vital bone was negatively correlated to connective tissue, tooth fragment, inflammatory infiltrate and non-vital bone. Non-vital bone was also negatively correlated to epithelium (Pearson correlation coefficient, Table 2).

### 3.4. Immunohistochemical Analysis

**Figure 4:**
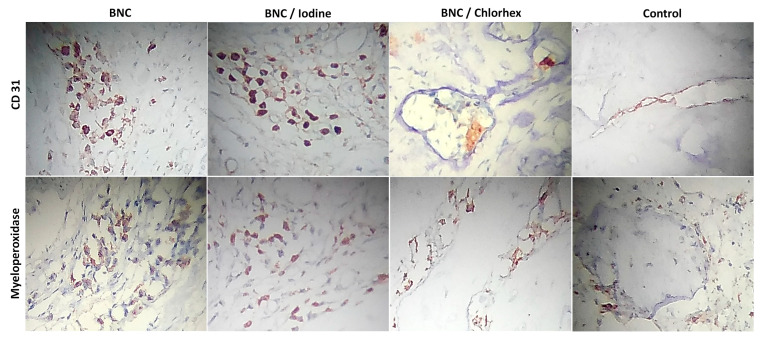
Immunostaining (400×) for CD31 and myeloperoxidase in BNC, BNC/Iodine, BNC/Chlorhex and Control groups. BNC = bacterial nanocellulose; Chlorhex = chlorhexidine.

The BNC/Iodine group showed greater CD31 immunostaining (median = 96.80 µm^2^) compared to BNC (median = 24.87 µm^2^) (*p* = 0.017) and Control (38.91 µm^2^) (*p* = 0.013), whereas BNC/Chlorhex (88.83 µm^2^) did not differ from any group (*p* > 0.05). Myeloperoxidase (MPO) staining did not significantly differ between the groups, with medians of 56.87, 55.22, 34.33 and 78.44, respectively, for BNC, BNC/Iodine, BNC/Chlorhexidine and Control (Kruskal–Wallis, Dunn’s multiple comparison test, Table 3, Figure 4). Because of technical problems, there was one missing case in the BNC/Chlorhex group in CD31 analysis, and in MPO analysis, there were two missing cases in the BNC/Iodine group, one missing case in the BNC/Chlorhex group and one missing case in the Control group.

### 3.5. SEM Analysis

**Figure 5:**
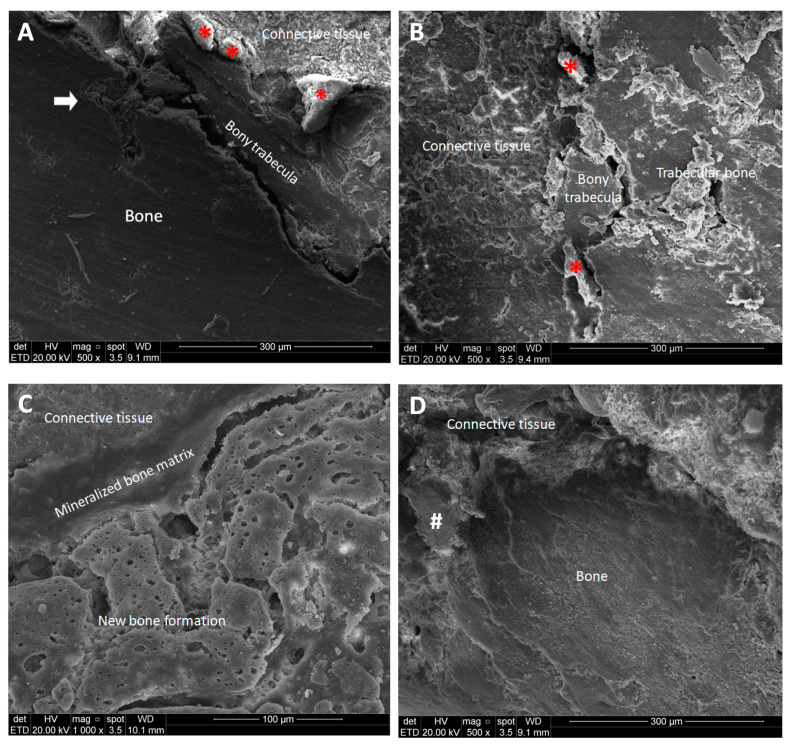
The boundary region between bone and connective tissue is shown in all groups. (**A**) BNC group: the arrow shows a small area of medullary bone and a small bony trabecula adjacent to an extensive area of bone with a smooth texture; adjacent to bony trabecula are structures compatible with osteoclasts (*). (**B**) BNC/Iodine group: areas of irregular surface surrounded by mineralized matrix with smooth surface characterizing the trabecular bone; small bony trabecula adjacent to connective tissue; adjacent to bony trabecula are structures compatible with osteoclasts (*). (**C**) BNC/Chlorhex group: several portions of mineralized matrix with resorption pit clusters characterizing new bone formation adjacent to an area of mineralized bone matrix. (**D**) Control group: rough surface bone and bony trabecula (#) surrounded by connective tissue. BNC = bacterial nanocellulose; Chlorhex = chlorhexidine.

**Figure 6:**
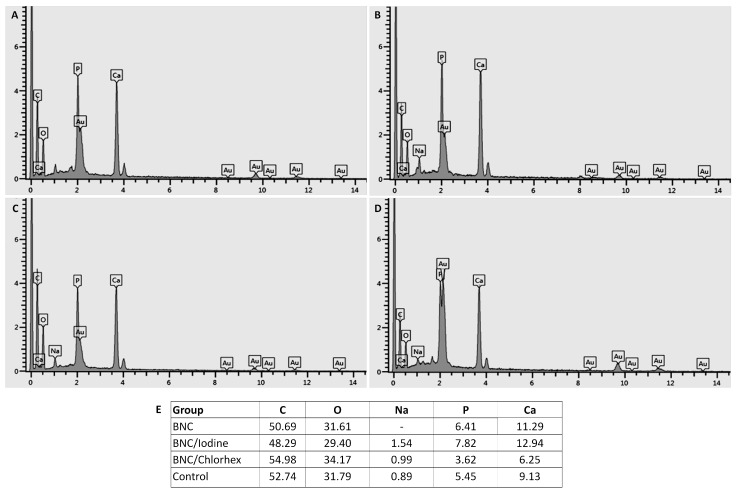
Mineral content of alveolar bone by energy dispersive spectroscopy (EDS) analysis. BNC (**A**), BNC/Iodine (**B**), BNC/Chlorhex (**C**) and Control (**D**) groups. Summary of findings in all groups ((**E**), %). BNC = bacterial nanocellulose; Chlorhex = chlorhexidine, C = carbon; O = oxygen; Na = sodium; *p* = phosphorus; Ca = calcium. The overlapping legends in (**D**) correspond to Au and P.

The groups of this study had similar findings on SEM analysis of alveolar bone, showing areas of mineralized bone and trabecular bone. Bone reorganization of the extraction area was observed as bony trabeculae (Figure 5A,B,D) and several areas of mineralized matrix with resorption pit clusters characterizing new bone formation (Figure 5C). Mineral content of the alveolar bone indicated the presence of carbon, oxygen, sodium, phosphorus and calcium (Figure 6).

## 4. Discussion

The present study investigated the macro- and microscopic effects of BNC alone or with chemisorbed iodine or chlorhexidine, on bone alveolar repair of tooth extractions in rats undergoing bisphosphonate therapy. On macroscopic evaluation, the frequency of oral lesions did not significantly differ between the groups testing BNC, with or without the antiseptics, and Control. Considering that all groups had undergone zoledronic acid therapy and tooth extractions, we expected better results in alveolar bone healing for those receiving BNC, regardless of antiseptic use. However, this did not happen in the macroscopic analysis. A possible explanation for this result could be the persistence of tooth fragments at the site of extraction of some animals in all groups, which could also interfere with wound healing. It is important to recall here that zoledronic acid increases bone density and makes tooth extraction more difficult. Corroborating such notion is the negative correlation found between tooth fragments and vital bone. Nevertheless, in the macroscopic analysis, the size of the oral lesions was still greater in the BNC/Iodine group compared to BNC/Chlorhex and Control, which was also not expected, since iodine was used in the attempt to improve the repair of the extraction wound [31,32]. Moreover, BNC/Iodine did not significantly differ from BNC regarding this variable. We could consider these results as suggesting that BNC does not improve alveolar bone healing under the conditions tested in our study. Accordingly, it was reported that despite the promising application of BNC membranes in soft-tissue repair, it did not induce bone repair in rat calvaria [33]. Nevertheless, that does not explain the results for its combination with iodine, which therefore requires the findings of the microscopic analysis, which we discuss next.

In histological analysis with H&E, even though some tendencies were observed, the greater proportion of connective tissue in the BNC/Chlorhex group compared to BNC was the only one showing statistical significance. Meanwhile, BNC/Chlorhex was the group with a tendency of having less vital bone, whereas vital bone had a negative correlation with connective tissue. It seems the larger amount of connective tissue in BNC/Chlorhex was a result of healing by means of less bone formation and more fibrous connective tissue, which would be related to chlorhexidine. Once again, BNC did not seem to improve wound healing, which was corroborated by the IHC and SEM results. However, some aspects concerning the properties of BNC and mechanism of action of the drugs tested need to be explored. Meanwhile, having greater lesion size on macroscopic analysis, BNC/Iodine had higher CD31 staining in IHC. One could point out that larger wounds would demand greater vascularization, since adequate blood flow is crucial for wound healing [34]. However, neither in the H&E parameters nor in MPO analysis did BNC/Iodine differ from the other groups. Such results seem conflicting. Anyway, it is important to consider that we used iodine in the form of povidone-iodine. The role of povidone-iodine in wound healing has been investigated with varying results [32]. It was reported that it can impair collagen synthesis, has a toxic effect on fibroblasts and keratinocytes and impairs epithelial cell migration, whereas other studies report healing improvement [32,35]. Wounds in mice showed earlier and complete neovascularization with povidone-iodine compared to other antiseptics [32], which corroborates our results for CD31. Also, better results for povidone-iodine compared to chlorhexidine, with faster healing of venous ulcers and burns, were reported [32]. Povidone-iodine, also called polyvinylpyrrolidone iodine, is a combination of molecular iodine and polyvinylpyrrolidone surfactant/iodine complex. It is a water-soluble complex comprising elemental iodine bound to a synthetic polymer in a 10% aqueous solution and the free iodine component. Povidone-iodine works as a reservoir of free iodine, which is the bactericidal agent [32,35,36]. In our study, we combined this complex to a further delivery system, which was BNC membranes, possibly interfering with the physicochemical properties of povidone-iodine. Dydak et al. [13], on the other hand, obtained good results testing BNC with chemisorbed chlorhexidine and povidone-iodine. However, it is important to remark that they performed an in vitro study, whereas ours used an animal model. Different environments determine different results. Regarding this point, it is known that povidone-iodine can have its activity reduced in the presence of organic matter such as certain proteins in wound exudates or body fluids [32,37]. Therefore, in vivo studies might be considered more clinically relevant, since in vitro studies do not take into account the host proteins neutralizing antiseptic activity [17].

Still, regarding the conflicting results of the BNC/Iodine group, some other considerations must be addressed. In IHC, CD31 is used primarily to evidence endothelial cells. Considering that angiogenesis is crucial for wound healing and that once the vessels are formed, they become part of the repaired tissue, the results for this marker in the BNC/Iodine group are plausible, as this group had larger oral lesions. MPO, on the other hand, which is classified as an inflammatory marker, did not show greater staining in the BNC/Iodine group. To understand this result, we need to point out some aspects regarding MPO. First, MPO is a key enzyme in neutrophils, which plays a significant role in the inflammatory response [38,39,40], being demanded especially in case of infectious injury and found to a lesser extent in monocytes, but usually lost during the maturation of these cells to macrophages [38]. At the wound site, its highest levels would occur at 24–48 h after injury, diminishing considerably afterwards. Second, MPO is a cationic compound, which reacts with halides such as chloride, iodide, bromide and thiocyanate, giving a potent antimicrobial agent. Considering the available iodine in the BNC/Iodine group, such reaction could have interfered with MPO levels. Third, human and murine neutrophils differ in MPO levels, which are 10–20% in murine compared to human cells. Finally, it is important to note the dual effect of MPO, exhibiting both a protective and harmful role in the maintenance and disturbance of tissue homeostasis [38]. In this regard, Zhao et al. [41] reported a protective role for MPO in bone homeostasis apart from its functions in inflammatory diseases. In sum, the conflicting results observed in BNC/Iodine, which, despite having larger oral lesion and greater CD31 staining, did not have greater MPO, could be related to the time of evaluation of our experiment (25 days after tooth extraction), the dual effect of MPO, the lower levels of MPO in murine/rat neutrophils and also the possibility of MPO reacting with iodine at the wound site.

We chose the concentrations of 10 and 0.12%, respectively, for povidone-iodine and chlorhexidine because these are the therapeutic concentrations commonly used in clinical routine. It is known that there is a lack of evidence around the concentration and formulation of antiseptics to be used in different kinds of wounds [32]. Moreover, authors report the paradoxical effect of 10% povidone-iodine, where iodine (the active compound) levels follow a bell-shaped curve and increase with dilution of povidone-iodine, reaching a maximum at approximately 0.1% strength solution and then decreasing with further dilution [17]. Solutions within 0.1–1% were reported to be more bactericidal than the 10% solution [32,42]. We tested BNC with chemisorbed iodine or chlorhexidine, one at a time, not both chemisorbed in the same membrane. Evidence suggests that povidone-iodine in combination with chlorhexidine may prove to be more effective, with a possible synergistic effect between them [17]. Concerning zoledronic acid administration, one could point out that it was a long-term treatment, subjecting the rats to a relatively long period of stress, which could interfere with the results. In this regard, it is important to recall that all the groups were subjected to the same time of treatment, avoiding biases when comparing the results between them.

Native BNC has no antibacterial activity, and it has been indicated for dressing chronic wounds. In our study, the animals did not have chronic wounds but a predisposition to having compromised post-extraction healing because of the bisphosphonate therapy. The inflammatory infiltrate in H&E and MPO in IHC did not show any significant difference between the groups, suggesting that the groups displayed a similar behavior regarding inflammation. It was reported that povidone-iodine can impair collagen synthesis and epithelial cell migration and has a toxic effect on fibroblasts and keratinocytes, thereby potentially having a detrimental effect on the healing process in non-infected human wounds. Some adverse effects of chlorhexidine such as cytotoxicity against eukaryotic cells affecting cellular viability and long-term tissue regeneration, induction of bacterial resistance, cross-resistance to antibiotics, potential triggering of anaphylactic reactions and risk of hydrolysis to carcinogenic 2-chloroaniline have also been reported [13]. However, we need to consider the time of analysis performed in our study, which would be enough for any recovery from possible damage caused by a single application of the drugs at a low dose. Moreover, we should point out that studies reporting cytotoxic effects used in vitro experimental models, which do not necessarily reflect clinical settings [31]. Therefore, further pre-clinical studies investigating the drugs with different posology could improve our knowledge of this theme. However, it is important to pay some attention to the referred toxic effects when choosing the posology of administration, especially in pre-clinical or clinical studies, and even more in clinical practice routine, to prevent any local or systemic damage.

According to the results of the present study, BNC with chemisorbed povidone-iodine is associated with increased vascularization in post-extraction wounds of rats under bisphosphonate (zoledronic acid) therapy, whereas BNC with chemisorbed chlorhexidine improves connective tissue formation. Although BNC, by itself, does not improve wound healing in these experimental conditions, it plays a role as an effective carrier for the antiseptics tested. These findings demand further studies including complementary histological techniques such as tartrate-resistant acid phosphatase (TRAP) and Masson’s trichrome, addressing not only different concentrations of the drugs, especially lower concentrations of povidone-iodine, but also both of them, iodine and chlorhexidine, chemisorbed on the same BNC membrane. Another aspect to explore in new studies is to compare BNC, combined or not with iodine and chlorhexidine, to materials already used at sites of tooth extraction such as collagen membranes, bone morphogenetic protein (BMP) and stem cells. Still, considering BNC membranes for use as scaffolds in alveolar bone surgery, studies investigating tensile strength, elasticity and compressive resistance are needed.

## Figures and Tables

**Table 1:** Histological analysis by using manual point counting technique (number of points).

	Group												
Variable	BNC			BNC/Iodine			BNC/Chlorhex			Control			*p* *
	Mean	SD	MD	Mean	SD	MD	Mean	SD	MD	Mean	SD	MD	
Epithelium	46.88	26.16	45.75	42.73	22.53	45.25	46.65	21.29	45.63	42.05	18.66	47.38	0.996
Connective tissue	130.03	31.95	131.25 ^A^	162.08	49.11	141.00 ^AB^	200.20	64.97	191.88 ^B^	161.60	38.56	165.75 ^AB^	0.048
Vital bone	282.60	60.95	281.88	286.80	68.94	264.25	239.33	93.89	214.00	316.35	83.27	295.13	0.218
Non-vital bone	54.48	49.94	54.50	35.90	44.86	29.25	42.55	26.18	49.50	23.40	34.62	3.63	0.273
Tooth fragment	14.83	24.77	0.63	18.08	29.90	0.00	19.15	21.83	7.50	20.40	23.75	8.00	0.757
Inflammatory infiltrate	11.90	11.24	10.50	6.93	8.02	4.38	15.05	13.71	18.00	6.35	8.36	3.25	0.438

BNC = bacterial nanocellulose; Chlorhex = chlorhexidine; SD = standard deviation; MD = median; * *p* = *p* value for Kruskal–Wallis. Medians followed by different letters in the row differ significantly (Kruskal–Wallis, Dunn’s multiple comparison test, α = 0.05).

**Table 2:** “*r*” for Pearson correlation coefficient.

Variable	Epithelium	Connective Tissue	Vital Bone	Non-Vital Bone	Tooth Fragment	Inflammatory Infiltrate	Size of Oral Lesion
Epithelium	1						
Connective tissue	−0.072	1					
Vital bone	−0.068	−0.547 **	1				
Non-vital bone	−0.359 *	0.018	−0.492 **	1			
Tooth fragment	0.053	−0.057	−0.388 *	−0.086	1		
Inflammatory infiltrate	0.152	0.197	−0.519 **	0.248	0.175	1	
Size of oral lesion	−0.023	−0.301	−0.016	0.271	−0.066	−0.002	1

* Correlation at 0.05 significance level; ** correlation at 0.01 significance level.

**Table 3:** Immunostaining (µm^2^) for CD31 and myeloperoxidase (MPO).

Group	CD31				MPO			
	MD	P25	P75	MR	MD	P25	P75	MR
BNC	24.87 ^A^	10.38	129.70	50.20	56.87	10.94	124.75	52.48
BNC/Iodine	96.80 ^B^	31.70	333.53	71.17	55.22	18.18	164.82	56.46
BNC/Chlorhexidine	88.83 ^AB^	18.78	194.00	65.96	34.33	6.46	93.94	46.31
Control	38.91 ^A^	14.40	92.90	49.37	78.44	23.58	202.15	63.19
*p* *	0.024				0.247			

BNC = bacterial nanocellulose; Chlorhex = chlorhexidine; MD = median; P25 = 25th percentile; P75 = 75th percentile; MR = mean rank; * *p* = *p* value for Kruskal–Wallis. Medians followed by different letters in the column differed significantly (Kruskal–Wallis, multiple comparison Dunn’s test, α = 0.05).

## Data Availability

The raw data supporting the conclusions of this article will be made available by the authors on request.
